# Letter Migrations between Words in Reading Aloud Can Result either from an Impairment in Orthographic Input or in Phonological Output

**DOI:** 10.3390/brainsci13040588

**Published:** 2023-03-30

**Authors:** Liora Toledano, Naama Friedmann

**Affiliations:** Language and Brain Laboratory, School of Education and Sagol School of Neuroscience, Tel Aviv University, Tel Aviv 69978, Israel

**Keywords:** developmental dyslexia, attentional dyslexia, Hebrew, migrations between words, phonological output buffer, orthographic-visual analyzer, reading

## Abstract

Letter migrations between words in reading aloud (e.g., reading “cane love” as “lane love” or “lane cove”) are known to result from a deficit in the visual-orthographic analysis and characterize attentional dyslexia. In spontaneous speech, individuals with impairment in the phonological output buffer may show migrations of phonemes between words. The purpose of this study was to examine whether migrations between words in reading aloud can also result from a deficit in the phonological output buffer, to explore the characteristics of migrations resulting from orthographic input and from phonological output deficits, and to examine methods to distinguish these two sources. Using tasks of reading aloud of lists of 92–182 word pairs, we identified 18 adults and adolescents with developmental dyslexia who made between-word letter migrations in reading aloud, significantly more than age-matched controls (372 adults, 26 7th-graders and 44 4th–5th-graders). To distinguish between the orthographic input and phonological output sources for these migrations, we administered a test assessing orthographic input without spoken output (written semantic decision on 140 migratable word pairs) and a repetition test of 36 auditorily presented migratable word pairs, assessing spoken output without orthographic input (as well as nonword repetition and 3 span tests). These tests indicated that the migrations in reading aloud of 10 of the participants with dyslexia resulted from an orthographic input deficit—they made migrations not only in reading aloud but also in written word pair comprehension, but not in word pair repetition. For the other 8 participants, the migrations resulted from a phonological output deficit: they made migrations in reading aloud and in word pair repetition, but not in comprehension, and had limited spans and made errors in nonword repetition. We identified several differences between the two types of between-word errors: first, the individuals with attentional dyslexia made omissions of a letter that appeared in the same position in the two words, but the phonological output buffer group did not make such omissions. Second, the groups differed in the origin of migration: orthographic input migrations involve letters that are orthographically adjacent, whereas phonological output migrations involve phonologically adjacent phonemes: phonemes that have just been spoken or that are prepared together in the phonological buffer for production. Migrations from the line below and from 2 lines above the target occurred only in the orthographic input group. This study thus indicates that between-word migrations in reading aloud can result not only from attentional dyslexia, but also from a phonological output buffer deficit, and offers ways to distinguish between the two.

## 1. Introduction

There are several types of dyslexia, a deficit in reading, each type resulting from a deficit in a different part of the reading process, and each showing different error types and properties. One type of dyslexia is attentional dyslexia, resulting from a deficit in the letter-to-word binding function in the orthographic-visual analyzer. The main characteristic of attentional dyslexia is migrations of letters between neighboring words in reading aloud, such as reading *clown frown* as “flown crown”. In this study we focus on between-word migrations and examine an additional possible source for these migrations in reading aloud: a deficit in the phonological output buffer. We then examine the different properties of between-word migrations of the two sources.

### 1.1. The Reading Process That Includes the Orthographic-Visual Analyzer and the Phonological Output Buffer

**Figure 1:**
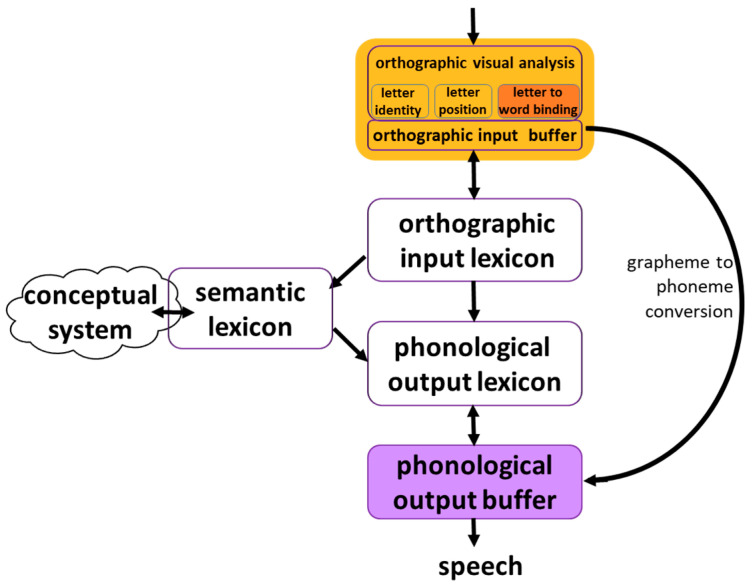
The dual route model of reading.

The cognitive model of word reading describes the process of reading—from a written word to its meaning and sound. The reading model that we assume here is the dual route model, as described in Friedmann and Coltheart [1], building on work by John Morton, John Marshall, Karalyn Patterson [2,3] and many others. It is the only model currently available that is able to explain a selective deficit that results in between-word migrations (as well as the different types of dyslexia currently known). This model is a multi-staged process, as presented in Figure 1. The process begins in an **orthographic-visual analysis** stage, which is responsible for letter identification, letter position encoding, and letter-to-word binding. The letter-to-word binding function, which is the focus of the current study, allows the reader to read letters within the words in which they appear and attenuate letters that appear in neighboring words [4].

The information then flows to an orthographic input buffer, which holds this information for a short time and parses the input string into graphemes and morphemes. This information proceeds in two routes: a lexical and a sub-lexical route. The lexical route connects an orthographic input lexicon and a phonological output lexicon. These lexicons hold orthographic and phonological representations of words that the reader already knows. The phonological representation then arrives from the phonological output lexicon in a **phonological output buffer** (POB), a short-term phonological memory component that holds all the phonological information until production, and assembles the phonological units into words and the morphemes into morphologically complex words. The POB is not only a component in the process of reading aloud, it is also part of speech production, in spontaneous speech, repetition and naming [5]: Spontaneous speech and naming use the route from the conceptual system, through the semantic lexicon, through the phonological output lexicon, to the POB; nonword repetition uses a route from the phonological input (not depicted in Figure 1) to the POB.

The lexical route also includes a branch connecting the orthographic input lexicon to the semantic lexicon and from it to the conceptual system, allowing for the comprehension of written words. The other route is a sub-lexical route, which converts graphemes (letters or letter groups, such as “sh”) into phonemes according to the grapheme-to-phoneme conversion rules of the language. The converted phonemes from the sub-lexical route arrive in the POB, where they are held and assembled.

### 1.2. Impairments Resulting from Deficits in the Orthographic-Visual Analyzer or the Phonological Output Buffer

Dyslexia is a deficit in reading. We work here under the theoretical and empirically-based assumption that dyslexia is a heterogeneous deficit. The reading process is a multi-component process, and each of these components may be selectively impaired. This results in various types of dyslexia, each resulting from a deficit in a different component (or connections between components) of the reading process, and creating different patterns of errors [1,6,7]. The various types of dyslexia may be acquired due to brain damage, in individuals who were reading normally before the brain damage; they can also be developmental, existing from birth and affecting the process of learning to read.

In the current study we focus on two types of impairments in reading aloud—an impairment in the orthographic-visual analyzer and an impairment in the POB. Although these two impairments are dramatically different, they share, as we will show below, a specific type of error that occurs when reading aloud: between-word migrations.

A deficit in letter-to-word binding, namely, in the ability to focus on one word and attenuate the words surrounding it, results in **attentional dyslexia**^1^, characterized mainly by migrations of letters between words [8,9,10,11,12,13,14]. Between-word migrations in attentional dyslexia preserve their within-word position, so if the first letter migrates, it migrates to the first position in the other word, and if the final letter migrates, it migrates to the final position in the other word. Migrations can result from words above, below or horizontally adjacent to the target word. Migrations occur more often when they create other existing words than when the migration does not create a word. Therefore, they occur more often in “migratable pairs”, word pairs in which between-word migrations (that preserve their within-word position) create existing words. Migrations are more frequent from the first to the second word than vice versa, and occur more often in the final letter than in the preceding letters. Several types of migrations occur in this form of dyslexia: the most well-reported one is a substitution, in which one letter from one word substitutes a letter in the other word (*coat goal* read as “coat goat”). However, impaired letter-to-word binding may also result in the omission of a letter that appears in the two words in the same position (*coat goal* read as “cat goat”) and additions (*lower fountain* as “flower fountain”). Because between-word migrations in attentional dyslexia result from a deficit in the early stage of the orthographic visual analyzer, which precedes the orthographic input lexicon and the access to the semantic system, migrations also affect written word comprehension (so the reader who made a migration would understand the written word as they read it, with the migration error [9]). Readers with attentional dyslexia make fewer errors when presented with single words [13] Developmental attentional dyslexia has been reported in English [14]), Hebrew [9], Arabic [22], French [23] and Italian [24], and even though not many articles report on it, it is relatively common between individuals with developmental dyslexia: in Hebrew for example, it is the fourth most common type of dyslexia [1,15].

A **deficit in the phonological output buffer** causes difficulties in all tasks involving phonological production: spontaneous speech, repetition, oral naming and oral reading [5]. Because the phonological output buffer is a phonological short term memory component, it has a limited capacity and is affected by the length of the phonological sequence: the more phonemes a sequence has, the harder it will be for the POB to keep the whole sequence, and the probability of errors increases [25,26,27].

The POB holds phonological units and assembles them, so a POB deficit causes omissions, substitutions and additions of phonological units of various sizes [28,29,30,31,32,33,34]. The assumption is that it holds phonological units of different sizes in separate mini-stores (phonemes, morphemes, number words and function words [31,35]). When the POB is impaired, errors occur within the mini-stores, leading to the substitution of units with other units of the same kind (e.g., substitution of one phoneme with another phoneme, substitution of one morpheme with another morpheme or omission of a whole morpheme).

The POB immediately follows the phonological output lexicon, and holding a sequence that exists in the phonological output lexicon is supported by activation from the lexicon. Nonwords are not represented in the lexicons and hence do not have such support. As a result, POB impairments cause a more severe deficit in nonwords compared to words in oral reading and in repetition [1,5,36].

One type of phonological error that is especially relevant for our current study is phoneme migrations between words. In developmental POB deficit, some of the POB-impaired individuals in Guggenheim’s study [37] made between-word migrations in reading aloud. Migrations are also reported for patients with output conduction aphasia, for whom the deficit is in the POB [25,26,38] and whose speech output is characterized by phonological errors. These errors include phoneme migrations within words [39], as well as migrations between words [40,41]. Individuals with typical language also make an occasional phonological error in speech—slips of the tongue [32,42,43]. Slips of the tongue include migrations of phonemes between words, which may also stem from a temporary failure at the POB level. In-depth analyses of these migrations [29,30,42,44,45] describe **anticipation** errors, in which a phoneme in the target word is substituted with a phoneme from a word that has not been produced yet (“slue sea” instead of *blue sea*), **perseveration**, in which a phoneme is substituted with a phoneme from a word that has been produced (“blue bea”), and **spoonerism** errors, in which the phonemes of two words are substituted with each other (“slue bea”). Most of these between-word migrations involve migrations of the first and final phonemes and, to a lesser degree, phonemes in the middle of the words [29,30,42].

### 1.3. Rationale of This Study

The considerations described in the previous section led us to hypothesize that when a person makes between-word migrations when reading aloud, these migrations may result from a deficit in the orthographic input stage (the orthographic visual analysis stage and the orthographic input buffer, which holds the products of this analysis) or from a deficit in the phonological output (the POB). Making this distinction also has important implications for treatment: manipulations that assist the patient in visually focusing on one written word would reduce errors that result from an orthographic input deficit [46], but would not necessarily help people with phonological output deficit. Conversely, dissecting the production of the phonological word into smaller phonological units [47,48] has been shown to assist individuals with POB deficits, but they are not expected to help individuals with orthographic input deficits. The aim of the current study is to examine whether indeed there are individuals whose between-word migrations in reading aloud stem from a deficit in the orthographic-visual analyzer and others whose between-word migrations result from a deficit in the POB.

A way to distinguish between these two sources of between-word migrations is to use tasks that isolate the orthographic input and the phonological output stages: tasks that assess the orthographic input and do not require phonological output, and tasks that assess the phonological output and do not use orthographic input.

Once the source of migrations of each participant is identified using such input and output tasks, we will further explore whether the properties of the migrations differ as a function of their source. Discovering the different properties of the two types of migrations would assist in the differential diagnosis of the two types of impairment. Furthermore, knowing more about the properties of these migrations would deepen our understanding of the nature and functioning of the orthographic visual analyzer and the phonological output buffer. We will focus on the following properties:

1. The type of between-word errors: do they involve substitutions of a letter/phoneme in the target word, additions, or omissions of the other instance of this letter/phoneme in the target word?
2. The origin of between word errors:
  a) Are there migrations from word pairs appearing after the target pair, or only from the other word in the pair or from a word pair appearing right before the target? The rationale for this comparison is that orthographic input migrations are expected to stem from words orthographically adjacent to the word, including those that follow the target word pair, but phonological output migrations are not expected to result from words that have not been in the POB yet—which have not been read aloud or prepared for production. Thus, phonological migrations are not expected to arrive from a word pair appearing after (below) the target;
  b) Are there migrations also from two lines above and below the target word or only from lines immediately adjacent to the target?
  c) Are the groups affected by different notions of adjacency? When two word pairs appear one above the other, the first word in the target pair is orthographically more adjacent to the word immediately above it (first word in the pair above), but phonologically, it is more adjacent to the word just pronounced, which is the second word in the pair above, which appears diagonally above the target;
  d) Direction of migrations: do more migrations occur from the first to the second word in the pair?
3. The position of the migrating letter within the word: Which within-word positions are more susceptible to migration?
4. The effect of the morphological status: is the migrating letter/phoneme part of the root or of a morphological affix? It has been demonstrated that the morphological structure of the word is available both at the stage of orthographic input (see [9,49,50,51,52,53] for findings regarding morphology in dyslexias in this stage, such as letter position dyslexia and neglect dyslexia) and in the phonological output buffer ([31]). Therefore, both stages may be affected by the morphological status of the migrating letter/phoneme.

## 2. General Method

### 2.1. General Procedure

Each of the participants performed the tasks in one or two sessions, in a quiet room- in the participant’s school or in our lab at the university. All sessions were audio-recorded. Each session lasted between one and two hours. In the tasks that required an oral response, the participant’s response was written down in real time by the experimenter, and was checked against the audio-recording and fully transcribed after the session by the two authors, as well as another experimenter. When a response was unclear, we listened to the recording again (and again). The very few cases of disagreement were discussed and an agreement was reached. The material in all tasks was in Hebrew. Within each test, the order of the items was the same for all participants.

### 2.2. General Statistical Analyses

The scores (typically the rates of errors of the various kinds) of each experimental participant were compared to the scores obtained by an age-matched control group using the (directional, one tailed) Bayesian single case-control score comparison test [54]. A dissociation within a participant between performance in two tasks was determined using the Bayesian statistical dissociation analysis [54,55]. Group-level comparisons between conditions, given normal distribution according to Kolmogorov–Smirnov test, were conducted using a paired *t*-test and repeated measure ANOVA. Comparisons between groups were conducted using *t*-test for independent samples and ANOVA, linear contrasts were also tested using ANOVA. When normal distribution could not be assumed, we used the Mann–Whitney test for the comparison between groups, and Wilcoxon’s signed rank test for comparisons between conditions within a group. FDR correction was applied [56]. The two-tailed alpha level used was 0.05.

## 3. Identifying Individuals Who Make between-Word Migrations in Reading Aloud

### 3.1. Participants in the Large Cohort from which the Individuals with between-Word Migrations Were Selected

The 18 participants with between-word migrations who participated in the current study were selected from a cohort of individuals with dyslexia who participated in experiments in the Language and Brain Lab at Tel Aviv University. They were selected to participate in the study if they made significantly more between-word migrations in reading word pairs aloud than their age-matched control group without dyslexia.

All of the participants in the large cohort were identified as having dyslexia using the TILTAN reading screening task [57]. The TILTAN screening test is designed to identify whether a reader has dyslexia, and if so, which type of dyslexia. To identify the various dyslexia types, the TILTAN screening test includes irregular words to identify surface dyslexia; migratable words to identify letter position dyslexia; morphologically complex words to identify all kinds of amorphia, deep dyslexia and orthographic input buffer dyslexia; function words to identify deep dyslexia and phonological output buffer impairment; long words for orthographic input buffer and phonological output buffer deficits; words with many orthographic neighbors for the identification of letter identity dyslexia and visual dyslexia; abstract and concrete words for deep dyslexia; words in which the substitution or omission of letters on one side creates other existing words for neglect dyslexia; and words in which the omission, substitution, addition or migration of a vowel letter creates other existing words for vowel dyslexia.

The TILTAN test comprises three lists: a list of 136 single words, a list of 40 nonwords, and a 30 word pair list (described in Section 3.2 below). The general performance in each list and the rate of each type of error were compared to age-matched controls (372 adults, 26 7th-graders, and 44 5th graders).

### 3.2. Procedure

To identify, within the dyslexia cohort, individuals who make between-word migrations in reading aloud, we examined the participants’ reading aloud of word pairs. We analyzed the errors in the TILTAN word pair test and administered two additional word pair lists for reading aloud. All the word pairs in the three tests were migratable pairs, i.e., the pairs are constructed such that each migration of letters between the words of the pair (maintaining the within-word position of the letter) creates another existing word. The words included in the three word-pair lists were verbs, adjectives and nouns, simple and morphologically complex words. An example for a migratable word pair included in the TILTAN word pair list is עננים גנבים, ɁNNIM GNBIM, pronounced: “ananim ganavim”, meaning ‘clouds thieves’; the migration of the third letter between these words would create ענבים גננים, ɁNBIM GNNIM, pronounced: “anavim gananim”, meaning ‘grapes gardeners’). The word-pair lists were presented on a white page, which was placed on the table in front of the readers until they finished reading all pairs on the page, without time limitation. Each page included 30–32 pairs, ordered one above the other, with a single space between the words in the pair. We focused on word pair reading rather than on reading within sentences and text because we wanted to avoid the effects of context on reading decoding errors (see [58,59,60,61,62]).

The lists were taken from three tests:

1. TILTAN reading screening test [57]: this test was built to identify types of dyslexia. It includes a single word reading list, a nonword reading list and a word pair list. In this section, we used the word pair reading part, which includes 30 pairs of 3–6 letter words (M = 4.5 letters), written in Arial font 14, with 1.5 line spacing between lines;
2. The word pair KISHBION test (the WOPI test): a test of 32 4–5 letter word pairs (M = 4.9 letters), written in Arial font 12, with 1 line spacing between lines;
3. The attentional dyslexia 120 pairs test (the AD120 test): this test includes 120 pairs of 2–5 letter words (M = 4.9 letters), written in David font 14, with 1.5 line spacing between lines.

All participants read the TILTAN test and all but three read the WOPI. Six participants read the AD120 test—four of them read only the first page of the test, which included 30 pairs. Participant SB read the TILTAN word pair and another word pair list, described in Section 5 (her performance in the second test is presented in the WOPI column, with a footnote). The performance of each participant in each task was compared to that of an age-matched control group. The 4th grader was compared to a 4th–5th grade control group, the 7th graders were compared to a 7th grader control group, and the adults were compared to an adult control group. The sizes of the control groups, as well as their performance, are presented at the bottom of Table 1.

### 3.3. Results: The Individuals with Between-Word Migrations Who Participated in This Study

Using the 3 migratable word pair lists, we identified 18 individuals who made significantly more between-word migrations than their age-matched controls without dyslexia. The percentage of between-word errors they made (as well as age and gender), as well as the percentage of between-word errors of the controls are summarized in Table 1. (Word pair reading tests also identified two additional participants who made between-Word migrations in reading aloud who showed deficits both in the input and in the output tasks, as well as additional deficits. We did not continue testing them, because the current study focused on the properties of selective deficits in the input or output.)

Our analysis was conservative in that it included only between-word migrations of consonants, as errors in one of the four Hebrew vowels can result from other kinds of reading difficulties, such as vowel dyslexia. Because earlier work on attentional dyslexia indicated that between word migrations are preserving the within-word position of the target word (e.g., the final letter migrates to the final position in the target word), we counted as between-word migrations only those migrations that preserved their within-word position.

**Figure 2:**
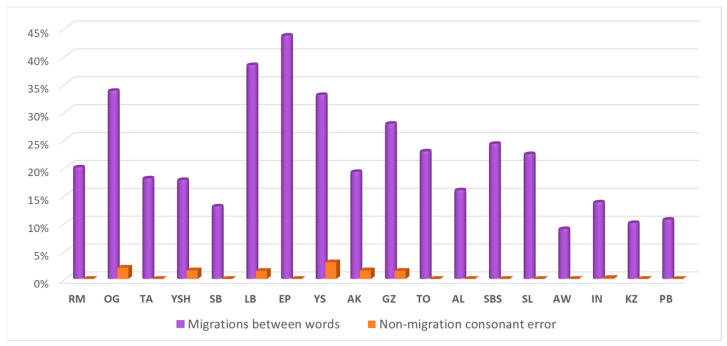
Percentages of consonant errors (substitution, omission, addition) that resulted from the neighboring words (migrations between words) in comparison to those that did not result from the neighboring words in all three word pair oral reading tasks.

The reading deficit of the 18 participants was specific to between-word migrations. The analysis of their consonant errors (substitutions, additions and omissions) that did not result from neighboring words in the three word pair reading tasks indicated that none of them made such non-between word consonant errors in a rate that was more than the controls. In all three tasks, for all participants together, counting all substitutions, additions and omissions, there were only 7 consonant errors that were not between-word migrations, out of a total of 2700 words, 0.2%. Namely, all 18 participants made many consonant errors that originated from the neighboring words, but none of them made other consonant errors that could not be accounted for by between-word migrations. The rate of substitutions, additions and omissions out of the number of word pairs each participant read in the three tasks combined, those that could and those that could not be accounted for by between-word migrations is summarized in Figure 2.

In line with Werth [63,64,65], who found that reading errors decrease when presentation/fixation time increases, Participant KZ, who read the TILTAN word-pair list very slowly, word by word, made very few errors on this list. However, her deficit manifested itself in the other tasks, in which she read at normal speed (this may indicate that we may have missed additional individuals with between-word migrations, who read very slowly and applied self-monitoring techniques to reduce errors).

All but one of the 18 participants who participated in the current study had a prior diagnosis of learning disabilities. They studied (in the present or in the past) in a special school for children with learning disabilities whose IQ is normal, or in regular schools with accommodations and special education teacher support for learning disabilities. The 55 year old participant did not have an official learning disability diagnosis (these diagnoses were not available when she was in school), but she reported struggling with reading throughout school, and that her children are diagnosed with a reading disorder. None of the participants sustained brain damage and none of them had neurological conditions (other than their dyslexia), so they were all cases of developmental dyslexia. All 18 participants were diagnosed with dyslexia on the basis of their performance in the TILTAN screening test [57]; All of them performed significantly below the control in the TILTAN test (using Crawford and Howell’s [66] and Crawford and Garthwaite’s [55] *t*-test, *p* < 0.01).

## 4. Distinguishing between Orthographic Input and Phonological Output Deficits: Tasks Assessing Orthographic Input without Phonological Output and Phonological Output without Orthographic Input

To establish, for each participant who made between-word migrations in reading aloud, whether their deficit results from an orthographic input or a phonological output deficit, we assessed their orthographic input and phonological output in isolation: first, we examined their phonological output buffer using word and nonword span tasks and a nonword repetition task, tasks that involve the POB.

Then, we examined whether the same participants made between-word migrations also in their repetition of auditorily presented word pairs, a task that involves phonological output but no orthographic input. Finally, we assessed orthographic input without phonological output using a semantic decision task on written word pairs.

### 4.1. Assessment of the Phonological Output Buffer Using Span and Nonword Repetition Tasks

#### 4.1.1. Procedure

1. Span tasks (FriGvi battery [26,67,68]): We administered three serial recall span tasks—two word span tasks and one test of nonword span. The short (basic) word span test included phonologically dissimilar 2-syllable words (for example: *derex kafe*, meaning ‘road coffee’). The long word span test included phonologically different 4-syllable words (for example: *limonada televizia*, ‘lemonade television’). The nonword span included 2-syllable nonwords, constructed by changing a single consonant in existing Hebrew words (for example: *lecax*, *demesh*, *kaxot*). The experimenter said each sequence at a one item per second rate and the participants were asked to recall the items serially. Each span test included six levels, each level included five sequences of 2–7 words or nonwords. The first level included 2-item sequences (level 2), and once the participant succeeded in recalling three sequences in a level, we moved to the next level, which included sequences that were one item longer. The span for each test was defined as the maximum level at which at least 3 of the 5 sequences were fully recalled, in the correct order; half a point was given for success in 2 out of 5 sequences in a level;
2. Nonword repetition task (BLIP [69]): The nonword repetition task is our most sensitive task for detecting a deficit in the POB. It includes 48 nonwords of 2–4 syllables, 4–9 phonemes: 24 of the nonwords were phonologically complex (e.g., example: *txicec*), and 24 were phonologically simple (e.g., *gelo*). The experimenter said each nonword separately, and the participants were asked to repeat it immediately. They were told that the items are not real words in Hebrew but rather words that we invented.

#### 4.1.2. Results

The results of the nonword repetition and the span tasks, summarized in Table 2, indicated that the participants who made between-word migrations in reading aloud could be classified into two different subgroups. One subgroup showed impaired nonword repetition and limited spans relative to the controls, indicating a deficit in the phonological output buffer^2^ (we call them below “the phonological output group”); the other subgroup’s performance did not differ from that of the controls in these tasks, suggesting that they had an unimpaired POB (we call them below “the orthographic input group”).

Table 2 presents all 18 participants who made between-word migrations in oral reading. They are presented in two groups—the top part of the table includes the 10 participants in the orthographic input group, who performed well on the nonword and span tasks, and not differently from their age peers. They were 12;3–28;11 year-olds, three females and seven males. The bottom part includes the 8 participants in the phonological output group, who performed below the controls in the nonword repetition task and in at least 2 of the 3 span tasks, five of them significantly so. They were 9;3–55;6 year-olds, four females and four males.

### 4.2. Assessment of Word Pair Production in Repetition

#### 4.2.1. Procedure

To examine the production of word pairs when no orthographic input is involved, we tested how the participants in the two subgroups repeat word pairs. A deficit in the orthographic input would cause migrations in reading but not in repetition of auditorily presented word pairs; a deficit in the phonological output would manifest itself in both reading aloud and repetition.

The word pair repetition task (the NADNEDA test [70]) included 36 word pairs. All word pairs were phonologically migratable, i.e., each position-preserving migration of a phoneme created a phonologically existing word. They were also orthographically migratable, like all word pairs described in Section 3 (for example: *xokeret sogeret*, meaning ‘female-researcher closing’; in which a migration of a letter/phoneme can create other existing words, such as *sokeret*, ‘scanning’). Each word was 2–3 syllable long, and 4–7 phonemes long (M = 5.9, SD = 0.8). All words were also migratable, so that a transposition of letters/phonemes within the word created other existing words (e.g., a transposition within the word above, *sogeret*, creates the word “soreget”, ‘knitting’).

The experimenter said a word pair out loud and the participant was required to repeat it immediately. The instruction was “I will say two words, there is no relation between these words. Please repeat them as accurately as you can”.

#### 4.2.2. Results

Table 3 summarizes the performance of the participants of the two groups in the word pair repetition task. The two subgroups showed very different performance patterns in this task: the phonological output group made between-word migrations in repetition, similar to their errors in reading aloud, whereas the orthographic input group did not make migrations in this task.

This supports the conclusion that the between-word migrations in the two groups have different sources, either the phonological output or the orthographic input.

### 4.3. Assessment of Word Pair Reading without Phonological Production

#### 4.3.1. Procedure

To examine whether between-word migrations occur also when reading word pairs without phonological production, we designed a semantic decision task on word pairs: the Watermelon task.

The Watermelon semantic decision task included 140 word pairs. Each pair was presented on the screen separately (Arial 28 font), for 1–2 s. Exposure time was determined individually for each participant in a preliminary training task in which the participants were requested to read word pairs on the screen using the same instructions as the Watermelon task but with different word pairs. They were asked whether the timing was comfortable for them. We started with 1 s in the preliminary task, and gradually extended the exposure time, each time in an interval of 250 ms, until the participant felt comfortable with the exposure time and reported they had enough time to read the two words.

There were two blocks in the task, a fruit block and a flavor block, each included 70 word pairs. In the fruit block, 58 pairs were constructed so that a position-preserving migration between the words created a name of a fruit/vegetable (a relevant example in English would be, dig fog), and 12 included a name of a fruit/vegetable (e.g., apple cupple). Similarly, the flavor task consisted of 58 pairs in which a position-preserving migration between the words created the name of a flavor (e.g., four soul), and 12 pairs included a name of a flavor (e.g., sweet dream).

Following the presentation of each word pair, a mask screen was presented (a picture of a heart made of fruits), and the participants were requested to say “yes” if they thought the preceding word pair included a word that is a name of a fruit or a vegetable (watermelon, fig, eggplant, apple, etc.), or a name of a flavor (sweet, sour, etc.). Sometimes, they also said the name of the fruit/flavor they saw, we did not prevent this. Once the participants made the decision and the verbal response, they could press the space key to see the next pair.

If a participant reported that they saw a fruit in the migratable non-fruit pair, this was taken as an indication that they had a between-word migration in silent reading.

#### 4.3.2. Results

Table 4 summarizes the performance of the participants of the two groups in the Watermelon semantic decision task. The two subgroups showed very different performance in this task: the orthographic input group made between-word migrations. These migrations made them identify fruit (or flavors) where there were no fruit (in word pairs in which a migration of letters between the words could create the name of a fruit/flavor). All but one of the participants in the orthographic input group made significantly more migrations in these pairs compared to the control group. In contrast, none of the participants in the phonological output group made between-word migrations in this task.

This supports the conclusion that the between-word migrations in reading aloud in the two groups have different sources. The orthographic input group makes errors in this task because their deficit is in an earlier component, affecting the orthographic visual analysis. As a result, their migrations precede the identification of the written words in the orthographic lexicon and the comprehension of these words, and therefore migrations affect their comprehension. The phonological output group, in contrast, is impaired in the phonological output and hence they do not err in this task, which involves only orthographic input stages and access from the orthographic input to the semantic system, but no phonological output.

After they said “yes”, the participants almost always said the name of the fruit/flavor they thought they detected. Although we determined their error rate only on the basis of their “yes/no” response, their naming of the illusory-conjunctioned vegetable/fruit/flavor was an indication that their error was indeed the result of a between-word migration.

### 4.4. Interim Discussion: Tasks Indicating Two Different Sources for between-Word Migrations in Reading Aloud

We used orthographic-only and phonological-only tasks to examine directly the orthographic input and the phonological output of the 18 participants who made between-word migrations in reading word pairs aloud. In the tasks that involved phonological output without orthographic input, 8 participants showed performance that was significantly below the controls. They made between-word migration errors in word pairs not only in reading aloud but also when they had to repeat auditorily presented word pairs. In addition, their word and nonword spans and their ability to repeat nonwords was significantly below that of the controls. The other 10 participants showed normal performance in these phonological tasks, but performed below the controls when they read word pairs, even when they did not have to read them aloud, in a semantic decision task. These results yield a distinction between two sources for between-word migrations in reading word pairs aloud: one is an orthographic input deficit, in letter-to-word binding, which affects both reading aloud and the comprehension of written words. The other is a phonological output buffer deficit, which affects the production of word pairs, both in reading aloud and in repetition of auditorily presented word pairs.

**Figure 3:**
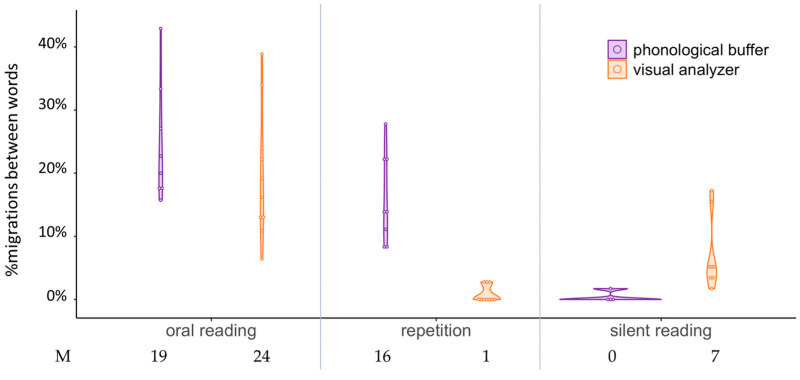
Percentages of migrations between words in word pairs in oral reading, silent reading (semantic decision) and repetition in the two groups.

Figure 3 summarizes the rates of migrations between words of the two groups in the different tasks involving word pairs: oral reading, repetition (phonological output without written input) and silent reading (semantic decision on written pairs without phonological output). The two groups had a large rate of between-word migrations in oral reading, both significantly higher than the controls (see Table 1 above), with an average of 19% between-word errors in the phonological output group and 24% in the orthographic input group, with no significant difference between the groups, t(16) = 1.03, *p* = 0.32, d = 0.52. They crucially differed in the tasks that involved only written input or only phonological output: The orthographic input group made significantly more between-word migrations than the phonological output group in the silent reading task (U = 31.5, *p* = 0.004), and significantly fewer between-word migrations than the phonological output group in the word pair repetition task (U = 0.000, *p* < 0.001).

The participants in the two groups present a double dissociation between reading without phonological output (comprehension of written migratable pairs) and phonological output without reading (repetition of migratable pairs). The dissociation was examined using the Bayesian criteria for dissociations in single-case studies (DissocsBayes_ES [54,55]). The difference between the cases’ standardized scores in comprehension and repetition was statistically significant on the Bayesian Standardized Difference Test for all participants (except OG who was only marginally impaired in the semantic decision task compared to the control group, *p* = 0.08, and his standardized difference was not significantly different from the controls).

All the participants in the orthographic input impairment group showed a classical dissociation between impaired comprehension of written migratable pairs and good repetition of migratable pairs, and all the participants in the phonological output impairment group showed a classical dissociation between impaired repetition of migratable pairs and good comprehension of written migratable pairs. The probability that the standardized difference for a member of the control population would be greater than that of the case (one-tailed) was smaller than 0.026 for all participants except OG.

**Figure 4:**
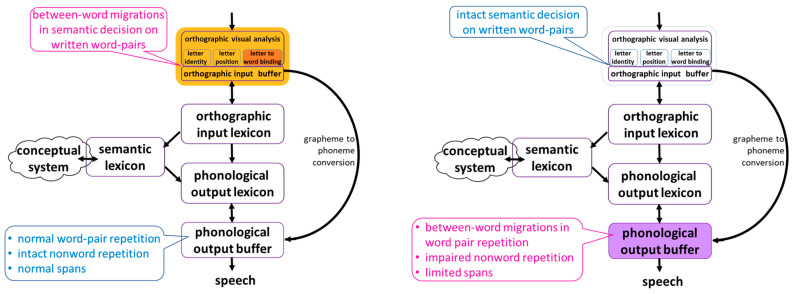
The impaired (cherry red) and intact (blue) performance in the input and output tasks of the participants of the two groups who showed between-word migrations in the oral reading of word pairs: the orthographic input deficit group (left), and the POB deficit group (right).

Figure 4 summarizes the impaired (cherry red) and intact (blue) performance of the participants of the two groups in the tasks described above, indicating their different loci of impairment in the word reading model.

## 5. Exploring the Properties of Input and Output Migrations

The properties of between-word migrations in attentional dyslexia were described by Friedmann, Kerbel and Shvimer [9]. Now that we identified an additional source of between-word migrations, the phonological output buffer, we turn to examine the properties of between-word migrations in the oral reading of the phonological output group and whether they differ from the properties of between-word migrations resulting from attentional dyslexia.

We examined the types of migration (substitution, omission, addition), the origin of the migration in neighboring words (the pair above or below), the origin of the migration within a pair (from the first to the second or from the second to the first word), the location of migration within the word (first, middle and final), and the effect of the morphological status of the migrating letter.

The analysis of the properties of between-word migrations was applied to all of the word pairs that each participant read (described in Section 3). In all of the analyses, we included only errors that were clearly between-word migrations and could not be classified as other errors (e.g., a migration that could also be classified as voicing error was not included in the analysis). Due to these strict guidelines, we analyzed only migrations of consonants because vowel errors could result from vowel letter dyslexia [71].

### 5.1. Types of Migration: No Omissions in the Phonological Output Buffer Group

Friedmann, Kerbel and Shvimer [9] describe three types of between-word errors in attentional dyslexia: substitutions, in which the target letter is substituted by a letter from a neighboring word (*bloom groom*-> “gloom broom”); omissions, in which one instance of a letter that appears in the same position in the two words is omitted (*store scare* -> “store care”); and addition, when the migrating letter is added to the target word (*rain boost* -> “brain boost”). To examine whether the same kinds of errors characterize between-word migrations resulting from a phonological output buffer deficit as well, we analyzed the rate of each kind of migration in the two groups.

#### Results

**Figure 5:**
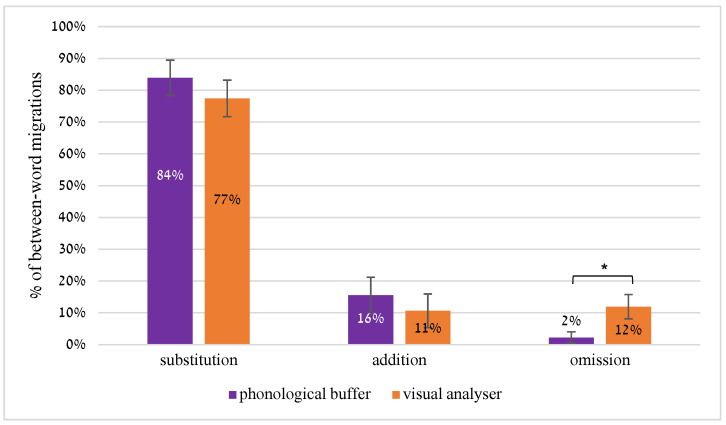
Percentages of between-word errors of the various types out of the total consonantal between-word errors. * *p =* 0.018.

The results, summarized in Figure 5, showed a striking difference in error types between the groups: whereas the orthographic input group made omission errors, the participants in the phonological output group practically did not make such omissions.

The omissions they did make, 2%, were of morphological affixes, so these errors could be ascribed to their POB deficit, which affects the reading and production of morphemes [31]. Once we exclude the morphological errors from the analysis and analyze only errors in the base/root, none of the participants in the POB group made any between-word omissions.

The groups differed significantly in the rate of omission errors (U = 14.5, *p* = 0.018), which remains significant with the FDR correction, [56]), but did not differ in the rates of substitutions and additions (t(16) = 1.05, *p* = 0.31, d = 0.52; t(16) = 1.21, *p* = 0.24, d = 0.61, respectively).

Thus, between-word errors that originate in the orthographic input include substitutions, additions and omissions, whereas between-word errors originating from the phonological output stage only include substitutions and additions, but, crucially, no omissions of the same letter that appears in the same within-word position in the two words.

### 5.2. Differences between the Groups with Respect to the Origin of Migration

We examined from which words the migrations originated, in two dimensions: migrations from lines above and below the target word (this section), and migrations within the pair (Section 5.3). The analysis of the origin of between-word migrations was applied to all the word pairs that each participant read (Section 3), as well as to another list of 64 migratable word pairs of 4–6 letters (M = 4.3 letters), and a list of 60 nonword pairs of 3–5 letter words (M = 4.0 letters), which were created for this study. These two tests were presented in the same way as the word pairs described in Section 3: The lists were printed in black font (Arial 12), on a white page, which was placed on the table in front of the reader for an unlimited time. Each page included 30–32 pairs, with 1 line spacing between lines.

#### 5.2.1. Migrations from Lines Below

In attentional dyslexia, migrations were found to originate in written words surrounding the target letter, including words above, below and horizontally adjacent to the target [9]. We hypothesized that this can be a point of difference between migrations originating in the orthographic input and migrations that result from a phonological output deficit. Migrations originating from the phonological output buffer should involve only words that the buffer holds, or words that the buffer has not completely discarded yet. This would mean that when reading aloud, migrations in the phonological buffer can originate in a word read previously or in another word that the buffer holds at the same time (if the buffer prepares the two words of the pair together for production), but crucially, not from a word in a line below that has not been spoken yet and is not prepared for production.

To examine this hypothesis, we analyzed, for each between-word error (of all kinds: substitutions, omissions and additions) that each participant made in reading all the word pairs, whether the letter (/phoneme) originated from a word above (in the two pairs above the target pair), below (in the two pairs below the target pair), or in the same pair as the target word. Cases in which the migration could have resulted from two different words (e.g., when the migrating letter existed, in the same position, both in the word above the target and in the word below it) were excluded from this analysis.

#### Results

The results indicated a clear difference between the two sources of between-word migrations, in line with our hypothesis: whereas the participants with orthographic input deficit had migrations from words above as well as below the target, the participants with the phonological output buffer impairment almost never showed intrusions from words below the target.

**Figure 6:**
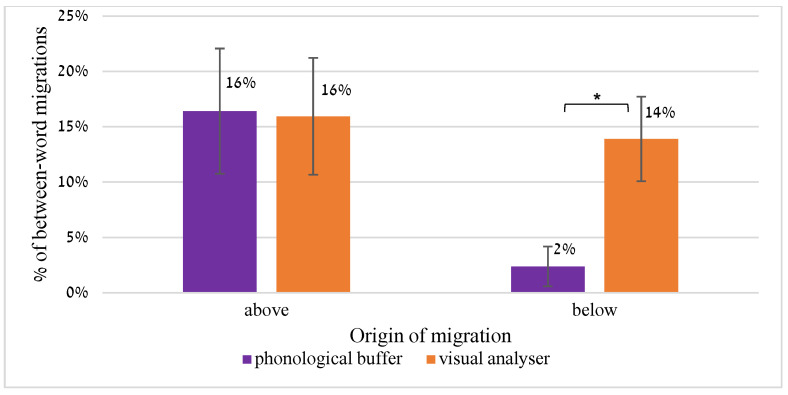
Origins of migrations between words out of all migrations between words: migrations originating in a word below or in a word above the target word. * *p* < 0.0001.

Figure 6 summarizes the results, showing the rate of migrations between words according to the origin of the migrating letter (out of the total number of between-word migrations).

The groups differed significantly in the rate of migrations from the words below the target (t(16) = 3.92, *p* < 0.0001, *d* = 1.96). Whereas for the orthographic input group 14% of the migrations originated in a word below the target, only 2% of the migrations of the phonological output group could be attributed to a word below the target.

Both groups made migrations from the words above the target (which have just been spoken) and from the other word in the pair, with no significant difference between the groups (neither for migrations from above the target word, t(16) = 0.09, *p* = 0.93, nor for migrations within the pair, t(16) = 2.05, *p* = 0.06). For both groups, most of the migrations originated from the other words in the pair and not from those above or below; this is probably a result of the way we constructed the word lists: the lists included migratable pairs, i.e., pairs in which a position-preserving migration creates other existing words, but they were not necessarily constructed so that each position-preserving migration from the words above and below the target would create an existing word. Still, the two groups read the same lists, but the orthographic input group made migrations from the words below and the POB group did not.

### 5.2.2. Migrations from Two vs. One Line Above

Another difference between the two groups related to the effect of words that are farther away from the target. When we compared migrations from words that were in the adjacent line (one line above or below the target pair) and from words that were two lines above or below the target pair, we found that all but one of the migrations in the POB group came from a single line above the target (and mainly, from the second word in this pair, see Section 5.2.3), with significantly more migrations from one line above than from two lines above, W = 36, *p* = 0.006. In contrast, in the orthographic input group, the migrations originated both from one and from two lines above/below the target: 67% of their migrations originated from the adjacent line, and 33% from two lines above/below the target (with a marginally significant difference between one and two lines above, W = 25, *p* = 0.06, and with no difference between the first and the second word in the target pair).

This makes sense, if two lines above and below the target are still within the orthographic-visual window, but only the phonological information of the previous pair is still present in the phonological output buffer. When we analyze only migrations from above the target word (because the POB group did not make migrations from below), and compare one and two lines above the target, we see that in the POB group, only a single migration came from two lines above (and 94% of the migrations originated from one line above), whereas in the orthographic input group, 26% of the migrations originated from two lines above.

### 5.2.3. Phonological vs. Orthographic Adjacency

**Figure 7:**
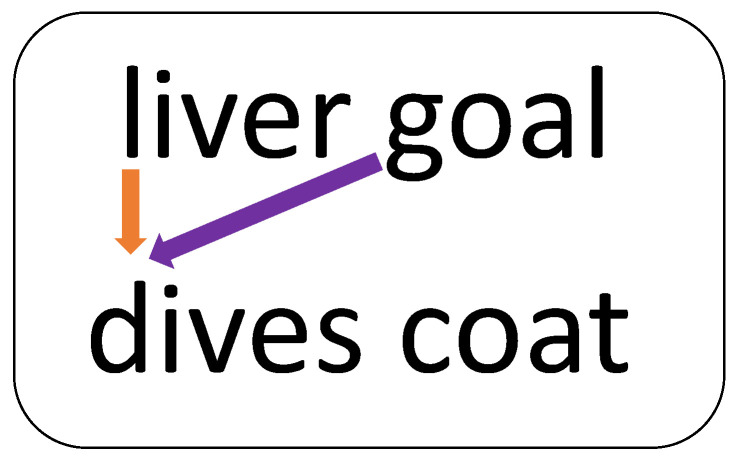
An example of vertical (orange) and diagonal (purple) possible migrations in two word-pairs placed one above the other. A vertical migration to the first letter of the target word *dives* creates “lives”; a diagonal migration would create “gives”.

A further finding that supports the different origins of migrations from the angle of orthographic adjacency vs. phonological adjacency relates to vertical vs. diagonal migrations. Have a look at Figure 7, demonstrating two pairs of words placed one above the other. When reading the first word in the second pair (“dives”), the first word in the pair above it is orthographically more adjacent; however, in the phonological sequence, the word that has just been pronounced, the second word of the pair above, is closer phonologically. Thus, for the first word in each pair, a vertical migration is closer orthographically, and a diagonal migration is closer phonologically.

In the analysis of migrations to the first word from the pair above, the orthographic vs. phonological distance created a clear difference between the groups: whereas the orthographic input group made 12 vertical migrations and only 2 diagonal migrations, the POB group made 2 diagonal migrations (and two vertical migrations that could be explained as a morphological or sibilant error rather than migrations). The groups differed significantly in the rate of vertical migrations (U = 19, *p =* 0.04, two tailed). For the second word in the target pair, a migration from a word placed diagonally is farther away from the one placed vertically, both orthographically and phonologically, and indeed such diagonal migrations did not occur, neither in the orthographic input nor in the POB group.

In our study, all the words were presented in word pairs listed one above the other. The difference is expected to be even more dramatic when the words are presented in text-like presentation. In this case no migration at all is expected from the line above in POB impairment, only in attentional dyslexia, an issue to examine in future studies.

Is it possible that the between-word migrations resulted from reading eye movement abnormalities? Clearly, for the phonological output impaired participants, this would not apply, because they did not have deficits in word pair reading when they did not need to read them aloud, and they had between-word migrations when they did not read. As for the individuals with orthographic input deficit, the analysis of their errors rules out such an explanation: had it been a deficit that causes incorrect location of fixation within the word, then we would expect all kinds of errors: letter substitutions, additions and omissions, and not only between-word migrations. However, as we reported above, such non-between-word migration errors were very rare and occurred only in 0.2% of the words. It is also interesting to note that Rayner et al. [14] reported that the eye movements of their attentional dyslexia patient were tested and found to be normal. It will be, however, interesting in future studies to examine directly eye movement patterns in the reading of individuals who make between-word migrations due to orthographic input deficits.

## 5.3. Characteristics That Apply to Both Groups

### 5.3.1. More Migrations from the First to the Second Word within the Pair

In attentional dyslexia, twice as many migrations occur from the first to the second word in the pair than from second to the first [9,72]. The same was found for skilled readers who read word pairs in short exposure durations [73,74]. We examined whether this property distinguishes between the two groups.

We analyzed migrations within all the pairs in the word pair lists, comparing migrations from the first to the second and from the second to the first word. We also examined whether migrations from above and below showed differences between the first and second word of the pair.

#### Results

There were significantly more migrations from the first to the second word, for both groups. In the orthographic input group, 57% of the migrations within the pair were from the first to the second word; in the POB group, 64% of the migrations within the pair were from the first to the second word. In the two groups together, first-to-second migrations were significantly more frequent than second-to-first (F(1,16) = 11.7, *p* = 0.003, η^2^ = 0.42), with no differences (no interaction) between the groups (F(1,16) = 1.72, *p* = 0.207, η^2^ = 0.097).

Interestingly, this pattern did not apply to migrations from outside the pair: where the first and second word did not differ significantly. In the analysis of migrations from word pairs above and below the target pair, in the orthographic input group there were (only) 45% migrations to the second word, and in the POB group, there were 52% migrations to the second word. These differences between migrations to the first and to the second word were not significant for either group (no significant main effect of word position, and no interaction, F(1,16) = 0.29, *p* = 0.60, η^2^ = 0.02).

Lumping together the two groups, in within-pair migrations, 60% of the migrations were from the first to the second word; in migrations from above and below, 48% of the migrations affected the second word.

### 5.3.2. Within-Word Position of Migrating Letters: More Errors in the Final Letter

In attentional dyslexia, the final letters were found to be more susceptible to between-word migrations than the middle and first letters [9]. Here, we examined whether the positions within the word that are most susceptible to between-word migrations are similar in the orthographic input and the phonological output groups.

To assess this question, we analyzed the consonantal position-preserving between-word errors in the word pairs presented in Section 3. We compared the rate of errors in the first letter, all middle letters and final letter. We calculated the number of migrations in each position out of the number of letters in this position in all word pairs whose migration creates an existing word. These amounted to 75 letters that could migrate and create an existing word in the first position, 48 in all middle positions, and 70 in final positions.

#### Results

**Figure 8:**
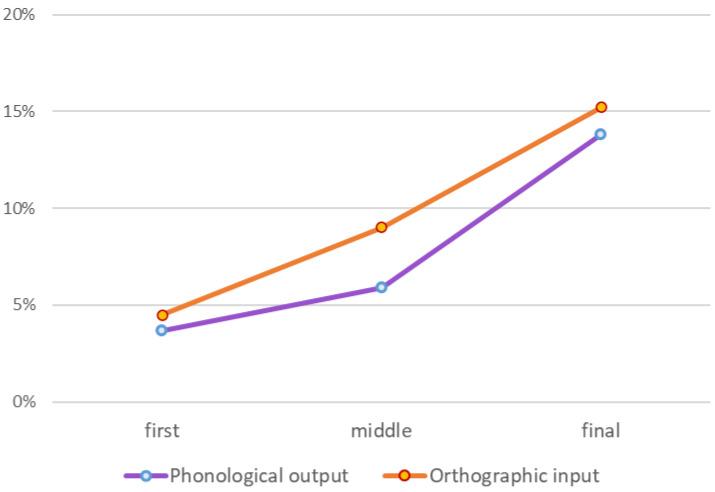
Percentage of consonantal migrations between words in first, middle and final positions.

Figure 8 shows the average number of consonantal migrations out of the number of word pairs with a lexical potential for migration in each position, for the two groups. These results show a linear increase in migration rates from the first letter, through middle letters, to the final letter (F(1,16) = 8.49, *p* = 0.01, η^2^ = 0.35). This tendency applied to both groups, with no difference (no interaction) between the groups (F(1,16) = 0.04, *p* = 0.96, η^2^ = 0.003).

The same pattern is revealed when we analyze only the morphological affixes or only the root letters (again, out of the potential for lexical migrations): for morphological affixes, both the phonological output group and the orthographic input group made far more errors in the final morphological letter than in the first morphological letter. In the phonological output group, there were 6% morphological migrations in the first letter, and 38% in the final letter; in the orthographic input group, there were 7% morphological migrations in the first letter and 45% in the final letter (all analyses were made out of the number of pairs that included morphological affixes in the relevant position). As for root letter migrations, the first, middle and final letters migrated in 3%, 4%, and 8%, respectively in the phonological output group, and in 0%, 7%, and 10% in the orthographic input group. This indicates that for the root letters too, the final position is the most susceptible to migrations. It also suggests that in orthographic input impairments, the first letter of the root does not migrate. Because the word pairs we used were not constructed to examine first root letter migrations, further studies should be conducted to answer this question directly.

### 5.3.3. Morphological Letters Are More Susceptible to Migrations Than Root Letters

Both groups showed three times more migrations of morphological letters (15% morphological migrations for the orthographic input group, 14% for the phonological output group out of the pairs that included morphological affixes that could migrate) than of letters that were part of the root (for both groups, 5% of the pairs that included root letters that could migrate). Of the 18 participants, 15 made more between-word errors in morphological letters than in letters of the root, and the difference between morphological and root migrations was significant in both groups (a significant main effect for the morphological status, F(1,16) = 13.11, *p* = 0.002), in line with Friedmann, Kerbel and Shvimer’s [9] findings. The main effect of morphological status applied for both groups, with no interaction (F(1,16) = 0.04, *p* = 0.84).

Interestingly, migrations between words that affected 2-letter morphological affixes (e.g., the plural feminine suffix -ות, -ot) were always of the whole affix, so the two letters migrated (or were omitted or added) together.

## 6. Discussion

Migrations between words in reading aloud are often ascribed to attentional dyslexia, a deficit in the orthographic input stage. However, work on speech production indicated that individuals with impairments in the phonological output make migrations of phonemes between words [29,30,40,41]. In this research we combined the two, and examined whether between-word migrations in reading aloud may also result from a phonological output deficit, and not only from a deficit in the orthographic input stages, and explored ways to distinguish the two.

We tested 18 adults and adolescents who made between-word migrations when they read aloud word pairs, and found that indeed there could be two separate sources for these errors. Ten participants showed migrations due to a deficit in the orthographic input, typical of attentional dyslexia. Importantly, however, eight other participants who made between-word migrations had a deficit in the phonological output, according to their impaired nonword repetition and limited recall spans.

To examine the sources of migrations in the two groups, we examined whether they made between-word migrations in reading without phonological output (using a semantic decision on word pairs), and phonological output without reading input (repetition of auditorily presented word pairs). The two groups crucially differed in these tests. The orthographic input group made between-word migrations in the semantic decision task but not in the repetition task; the phonological output group made between-word errors in the repetition task but not in the semantic decision task.

We concluded that indeed there are two types of between-word migrations in reading aloud: orthographic input migrations, which occur in the early stages of orthographic-visual analysis and affect the further comprehension of the written words; and phonological output migrations, which affect the phonological output even when there is no written input. Orthographic input migrations result from attentional dyslexia, a deficit in the orthographic-visual analyzer (or possibly in the orthographic input buffer), in the letter-to-word binding function; phonological output migrations are a result of a deficit in the phonological output buffer, which is not attentional dyslexia, but rather a different kind of deficit, which also affects word and nonword production outside the context of reading. As far as we know, this is the first report of the effect of an impairment in the phonological output buffer on the reading of word pairs^3^.

The finding that there are two separate sources for between-word migrations also bears on the question of the unitary vs. multi-factorial nature of developmental dyslexia, and on the issue of the deficit underlying developmental dyslexia. Our findings support the view of different types of dyslexia, resulting from deficits in different loci in the reading process and manifested in different error types and different properties (see also [1,6,7,75]). They also join previous research [58,71,76,77,78,79,80,81,82,83,84,85] to show that not all individuals with developmental dyslexia have a phonological deficit (in contrast with claims suggesting that what underlies developmental dyslexia is a phonological or phonological awareness deficit [58,71,86,87]). Ten of our 18 participants who made many between-word migrations showed completely normal phonological abilities, as reflected in their good nonword repetition, good word pair repetition and normal word and nonword memory spans.

### 6.1. Differences between Orthographic Input and Phonological Output Migrations between Words

We continued to analyze the properties of between-word errors of the two sources in reading aloud of word pairs and found two main differences between the two. The first was the type of error: both groups made between-word errors of substitution and addition (substitution—a migration of a letter from one word, substituting the letter in the same position in the other word; and addition—migration of a letter from one word, that is added to the same position in the other word). The two groups crucially differed in another type of between-word error: **omission errors**. Only individuals with orthographic input deficit made omissions of letters that appear in the same position in the two words (such as the omission of “l” in clean b**l**each -> clean beach). The individuals with a phonological output deficit never omitted such letters (or actually phonemes). This may result from the difference in the source of errors in the two groups: in the orthographic input, the only difference between the two same letters that appear in the same within-word position, is their appearance in different words. A patient who cannot bind a letter to the word in which it appears, would not be able to distinguish between the two instances of the same letter and may hence omit one of the instances (see [9] for a discussion of this error along the lines of Leibniz’ identity of indiscernible principle [88], and [58] for a similar discussion with respect to letter position dyslexia). Individuals with impairment in the phonological output buffer, in contrast, have difficulties holding phonemes and ascribing them to the correct word. In this case, a phoneme appearing twice in the same within-word position would only strengthen the activation of this phoneme, and increase the probability of it being produced correctly (for relevant discussions of repeated letters, see [45,89].

The other difference between the groups pertained to the **source of migration**: orthographic input migrations involve letters that are orthographically adjacent, whereas phonological output migrations involve phonemes that have just been spoken or that are prepared together in the phonological buffer for production. This was reflected in several findings of this study.

First, whereas orthographic input migrations occurred both from the word pairs above and from the word pairs below the target word pair, phonological output errors only originated in the word pair above, and never from the lines below. This makes sense: when the source of the error is orthographic-visual in nature, both the lines above and the lines below the target are in the orthographic attentional field and affect reading. However, in the phonological output buffer, only words that have just been produced and words that are prepared together to be produced may affect the buffer, namely, previous words and words within the word pair, but not words in the next line, which have not reached the phonological output buffer yet. Therefore, words in the next line do not affect individuals with phonological output buffer impairment.

A related finding was that in the orthographic input group, migrating letters came both from one line above the target and, to a lesser extent, also from two lines above. Phonological output migrations, in contrast, never resulted from two lines above.

Finally, when presented with a list of word pairs one above the other, the orthographic input migrations mainly came from words that were visually closer, appearing immediately vertically above (or below) the target word (e.g., letters from the first word in the pair above migrated to the first word in the target pair). In contrast, the phonological output migrations originated from phonologically closer words and hence came from the word that had just been produced, namely, the first word in a pair received phonemes from the second word in the previous pair (situated diagonally from it).

### 6.2. Similarities between Orthographic Input and Phonological Output Migrations between Words

The analysis of between-word errors in reading aloud of word pairs also allowed us to learn more about the properties of migrations that applied to both groups:

(a) There were significantly more errors toward the end of the word: more migrations in the final letter than in the middle letters and more migrations in the middle than in the first letter. This may indicate that letters in the beginning of the word are better bound to the word in which they appear, and that this letter-to-word binding works serially from the beginning to the end of the word. This applied both to morphological letters and to root letters. Interestingly, the orthographic input group did not have any migrations in the first root letters—this may indicate the superior status of the first letter of the root in orthographic-visual analysis. The importance of the first letter of the root may result from its special status in lexical access;

(b) There were more migrations within word-pairs from the first to the second word, in line with previous findings from attentional dyslexia [9,72]. This tendency seems to characterize skilled reading in short exposure durations [73,74], and may be related to the unidirectional nature of both reading and phonological output processes [45]. Migrations from word pairs above and below the target word pair did not show this effect, probably because a word in a pair below (or above) and the target word do not form a natural orthographic or phonological unit in which such directionality applies;

(c) There were more migrations of letters that belonged to the morphological affixes than of letters that were part of the root, in line with Friedmann, Kerbel and Shvimer’s findings [9]. This analysis was carried out only out of the relevant pairs—morphological migrations were calculated out of the pairs that included two different morphological affix letters in the same position, and root migrations were calculated out of pairs in which a migration of a root letter creates an existing word. This means that the reason cannot be a difference in lexical potential for the two migration types, but that both the orthographic-visual analysis stage and the phonological output buffer are sensitive to the morphological structure of the words. This finding of more migrations of morphological affixes than root letters indicates the difference in status between the root and morphological affixes. It also indicates that the morphological status is already available very early on in the orthographic-visual analysis stage, as has been shown in several types of dyslexia in this stage [9,49,50,51,52,53]. The morphological structure is also available at the phonological output buffer stage [31].

### 6.3. From the Results to Differential Diagnosis

These results have immediate implications as to how we can distinguish between the two sources of between-word errors. When a patient makes between-word errors in reading word pairs aloud, to distinguish between a deficit in the orthographic input and a deficit in the phonological output, one can:

1. Test the patient’s repetition of the same word pairs, presented auditorily. Between-word errors that also appear in repetition indicate a phonological buffer deficit;
2. Test the silent reading of word pairs (e.g., semantic decision of written word pairs). Migrations that affect comprehension indicate a deficit in the orthographic input;
3. Examine error types: If the patient makes omissions of letters that appear in the same position in the two words in the pair, the deficit is in the orthographic input;
4. Examine the source of migrations: if letters migrate not only from above and from within the pair, but also from the pairs below, the deficit is in the orthographic input. Migrations from two pairs before or after also characterize orthographic input deficit. Additionally, diagonal migrations from the second word in the pair above to the first word in the target pair characterize phonological output deficit; vertical migrations from the word immediately above or below the pair characterize orthographic input deficit.

One should bear in mind that some individuals may be impaired in both the orthographic input and the phonological output. In this case, they are expected to make migrations both in repetition and in semantic decision, and to have omissions and migrations from word pairs below. Therefore, it seems best to conduct both the repetition and the semantic decision tasks. Additionally, it is recommended to administer a long enough list of migratable word pairs (above 100), to enable the analysis of error types and sources of migrating letters.

### 6.4. Theoretical Implications

Our results shed some more light on the letter-to-word binding function in the orthographic-visual analyzer. Early accounts of this function, such as Shallice and Warrington, for example, who discovered this dyslexia [12], related to it as an attentional function, in the frameworks of Treisman’s feature binding theory [90] and Posner’s attentional spotlight [91]. Shallice later discussed the attentional source of this deficit in more detail, and suggested that the deficit lies in the “control system concerned in the parallel-to-sequential translation process” (p. 311, [92]), an account later adopted also by Warrington, Cipolotti and McNeil [93]. He suggests that the binding function sets an attenuation filter of the desired size (a single word, in text reading), which reduces the output of the letter-level analysis outside the appropriate window. An impairment in the control mechanism that applies this filter or in determining the size and locus of the attentional window results in attentional dyslexia, causing letters from outside of the word (from outside the required attentional window) to be activated together with letters inside the window. Saffran and Coslett [13] adopt this approach. Ellis and Young treat this function as a function that groups perceptually letters of the same word (p. 224, [4]) (essentially, Shallice’s account focuses on the suppression of the words around the target word, and Ellis and Young focus on the grouping of letters in the target word).

Such approaches treat attentional dyslexia as a result of a general visuo-spatial attentional deficit. These approaches need to be modified in light of the double dissociations found between attentional dyslexia without attentional deficits (attentional dyslexia without selective attention deficit, without attention control deficit, without orienting attention deficit and without sustained attention deficit, and attentional deficits without attentional dyslexia [15,16,17]). If one wants to hold on to the attentional accounts, one can think of it as an orthographic-specific attentional deficit, of an attentional mechanism harnessed to the reading process. Additionally, these accounts relied on data both from (a few) individuals with acquired attentional dyslexia and from healthy skilled readers who read word pairs under short exposure durations [73,94,95,96]. However, it seems that the between-word migrations in attentional dyslexia and in skilled readers result from malfunctioning at different stages of the reading process: for attentional dyslexia, it is in the early stage of orthographic-visual analysis; for healthy readers under short duration, it is at the level of the orthographic input lexicon, or the access to it [74]. Thus, data from healthy readers cannot inform the characterization of the letter-to-word binding mechanism that is affected in attentional dyslexia.

Our results, together with Friedmann, Kerbel and Shvimer’s [9], may shed some light on the functioning of this mechanism, from the pattern of impairments shown by our ten individuals with a deficit in the letter-to-word binding mechanism, and by the ten participants in Friedmann, Kerbel and Shvimer [9]. The results indicate that letter-to-word allocation (or “grouping” in Ellis and Young’s terminology) functions serially from the beginning of the word to its end, and from the first to the second word. It seems that the orthographic-attention spotlight is the strongest in the beginnings of words (in comparison to the ends of words), and in the first words (in comparison to the second words). This can be seen in two findings in the current study: more between-word migrations occurred in the ends of the words, and more letters migrated from the first to the second word within a pair. A finding from [9] that is also consistent with this view is that longer words yielded more between-word migrations. The findings of the current study (as well as in [9]) also indicate that root letters migrate less than morphological affix letters, which may suggest that the binding mechanism is more “attentive” to root letters, due to their relative importance.

The attenuating window seems to be applied to words before (here—the lines above) and to words that appear after (the lines below) the target word pair, as well as to the other word in the pair. This can be seen in the finding that when this window fails, we see migrations from word pairs above and below the target word. Migrations also occur from the other word in the pair, and these occur at a higher rate. Thus, attenuation seems to be reduced in words that are part of the same “reading unit“—more errors occur within a word pair than from words around the target. This was found in the current study (more migrations from the other word in the pair than from the pairs above and below; recall, however, that the tests were created with migratable word pairs but were not designed so that vertical migrations would always create existing words), and the Friedmann, Kerbel and Shvimer [9] study shows that it is not a vertical vs. horizontal distinction: in the Friedmann, Kerbel and Shvimer [9] study, when the pair was presented vertically, one pair at a time, it yielded exactly the same rate of between-word migrations as did the word pairs presented horizontally. It may be ecologically reasonable for the binding/suppression mechanism to be more lenient when it comes to words that need to be read together: when reading a sentence, information from words before and after the target is useful. Information from lines above and below may be less useful and hence, the attenuation mechanism may be fiercer against them. Still, the fact that migrations occurred from one and two lines above and below, we can conclude that the orthographic attention window for letter-to-word binding has an approximate size of 2 words of each side around the target word.

The results also shed some light on the functioning of the phonological output buffer. Specifically, the results indicate that the phonological output buffer holds and prepares more than one word at the same time. This is supported by the general finding of migrations between words in the phonological output buffer, and more specifically in the finding that migrations in the phonological output buffer also occur from the next word, which has not been said yet, to the first. More generally, there are indications that the buffer has to hold more than one word for the sake of stress placement in the sentence level, prosodic modification and more. Therefore, the buffer has a difficult task: it has to hold more than one word, and make computations on more than one word, and at the same time, it has to keep the borders of each word, in order to prevent migrations between words, somehow similarly to the “attentional window”, described for orthographic input.

Finally, we found that when morphemes migrate, they migrate as a whole—the whole multi-letter or multi-phoneme morphemes migrate between words. This is consistent with the view of the phonological output buffer, as we described in the Introduction, as holding and assembling phonological units of various sizes. It will be interesting to examine in further research whether morphological templates and function words migrate as a whole in cases of phonological output buffer deficit, or whether phonemes of these units can migrate separately.

### 6.5. Clinical Implications

Beyond the theoretical contribution of these findings, suggesting that between-word migrations in reading aloud may result not only from attentional dyslexia but also from a deficit in the phonological output buffer, the results also have direct clinical implications.

First, the results suggest that a patient who makes migrations between words in reading may have a deficit in orthographic input stage, but they may also have a deficit in the phonological output buffer, and their deficit should be assessed, for example using nonword repetition, span tasks and nonword reading.

In addition, the differential diagnosis that our findings enable is important for treatment, as different impairments require different treatment approaches. A treatment approach in which the reader hides the words above and below the target word in text reading is expected to help only readers with attentional dyslexia, who experience migrations from lines above and below. This manipulation is not expected to affect individuals with a POB impairment because in text, the words in the line above have long been out of the POB, and the line below has not entered the POB yet. Similarly, large spacing between words has been shown to assist some readers with attentional dyslexia, but it is not expected to affect those whose between-word migrations result from a POB impairment.

Another finding of [46], which examined various text manipulations that reduce errors in attentional dyslexia, was that presenting each word separately, for example using a word-sized reading window cut in a piece of cardboard and placed around a single word each time, which also hides words immediately before and after the target word within the text significantly reduced the rate of between-word migrations. This manipulation is effective when the deficit is in the orthographic input, and it would be interesting to examine in future studies whether it also reduces errors in POB impairments, because it may create a situation in which each word is prepared for production separately.

Other than that, there are treatment directions that have been proven effective for phonological output buffer impairment [47,48], and it would be interesting to test whether these treatments also reduce between-word migrations in reading aloud in this population.

The findings of this study also help in predicting what else will be difficult for the patient and what should be unimpaired. For example, here we tested word and nonword reading, rather than sentence and text reading. However, between-word migrations of the kind we described here would also affect sentence and text reading. For individuals whose between-word migrations result from orthographic input, the impairment would also affect the comprehension of written word pairs and of written text. In contrast, individuals whose migrations result from a phonological output buffer impairment should have unimpaired comprehension of written text, if they read silently and not aloud.

## 7. Conclusions

This study is the first to describe two different sources for between-word migrations in reading aloud: a deficit in letter-to-word binding in the orthographic visual analysis stage, and a deficit in the phonological output buffer.

We identified several aspects in which these two differ: firstly, the orthographic input deficit affects not only reading aloud, but also the comprehension of written words, in contrast, the phonological output deficit does not affect comprehension but it does affect other cases of spoken production, including word and nonword repetition. Furthermore, we found a major difference between the groups with respect to error types: only the orthographic input group but not the phonological output group omitted letters that appeared in the same position in two neighboring words. The groups also differed with respect to the properties of between-word migrations. Notably, the orthographic input migrations were affected by orthographic distance, and resulted from words above, below and next to the target word, whereas the phonological output errors were affected by phonological distance and did not stem from words below the target.

Beyond the new tasks and analyses that this study suggests for distinguishing between the two sources of between-word migrations, it also bears on the theoretical understanding of the letter-to-word binding function in the orthographic-visual analyzer and of the phonological output buffer.

## Figures and Tables

**Table 1:** The three word-pair reading aloud tests: %migrations between words out of the number of word pairs the participant read.

Participant	Age	Gender	%Migrations between Words Reading Words Aloud		
			TILTAN	WOPI	AD120
RM	9;3	M	23	−	17 ^b^
OG	12;3	M	37	22	43
TA	12;6	F	17	19	−
YSH	12;8	M	10	25	−
SB	12;11	F	13	11 ^a^	−
LB	13;6	F	43	34	−
EP	13;8	M	23	63	−
YS	13;11	M	33	−	−
AK	14;2	M	13	25	−
GZ	14;3	F	17	38	−
TO	14;6	M	27	19	−
AL	14;10	M	3	28	−
SBS	15;5	F	17	31	−
SL	22;6	M	10	34	−
AW	23;11	M	7	6	14
IN	27;11	M	3	34	11
KZ	28;11	F	3	28	7
PB	55;6	F	13	9	10
Control group 4th–5th grade: M (SD)			4.4 (4.6)		
N			44		
Control group 7th grade: M (SD)			2.1 (2.8)	3.4 (3.2)	2 (2.6)
N			26	21	7
Control group Adults: M (SD)			1.8 (2.6)	1.4 (1.8)	2.4 (2.8)
N			372	20	130

Shaded cells indicate migration rates that were significantly higher than the age-matched control. A minus sign (−) indicates that the participant did not read the list. ^a^ SB read a different word list instead of the WOPI: the list described in Section 5; ^b^ RM’s performance in the AD120 test was compared to a 7th grade control group.

**Table 2:** Performance in the BLIP nonword repetition test (%correctly repeated) and in the FriGvi phonological working memory serial recall tests (spans).

Group	Participant	Nonword Repetition	Spans		
		% Correct	Short Words	Long Words	Non-Words
Orthographic input (N = 10)	EP	94	5	4	3
	LB	94	5	4	3
	OG	96	4	4	3
	TO	92	5	4	3
	AL	94	5	4	3
	GZ	92	5	4	3
	IN	98	5.5	4.5	3
	AW	92	4.5	4	3
	KZ	92	5	4	3
	SL	94	6	5	3
Phonological output (N = 8)	RM	71	4	3	3
	TA	67	4.5	3	2
	YSH	75	4	3	2
	SB	65	4.5	3	2
	YS	48	−	−	−
	AK	60	4	3.5	2
	SBS	75	4	3	2.5
	PB	83	4.5	4	2.5
Control group 7th grade: Average (SD) N		94 (3) 18	5 (0.5) 28	4.1 (0.5) 28	3.1 (0.4) 28
Control group Adults: Average (SD) N		95 (3)	5.3 (0.7)	4.3 (0.6)	3.3 (0.5)
		20	173	69	187

Dark shaded cells denote performance of a participant in a task that is significantly lower than the matched control using Crawford and Howell’s [66] *t*-test; lightly shaded cells mark performance that is lower than the matched control, but not significantly. A minus sign (−) indicates that the participant did not participate in the test.

**Table 3:** Word pair repetition (NADNEDA test): Percentage of migrations between words out of the number of word pairs the participant repeated.

Group	Participant	%Migrations between Words Auditory Word Pair Repetition
Orthographic input (N = 10)	EP	3
	LB	3
	OG	0
	TO	0
	AL	0
	GZ	3
	IN	0
	AW	0
	KZ	0
	SL	0
Phonological output (N = 8)	RM	14
	TA	14
	YSH	11
	SB	22
	YS	28
	AK	8
	SBS	22
	PB	8
Control group 7th grade: Average (SD)		2.3(2)
N		20

Shaded cells indicate migration rates of a participant that are significantly higher than the control group using Crawford and Howell’s [66] *t*-test.

**Table 4:** The Watermelon semantic decision task: percentage of migrations between words.

Group	Participant	%Migrations between Words Semantic Decision
Orthographic input (N = 8)	EP	5
	LB	16
	OG	2
	GZ	3
	IN	5
	AW	3
	KZ	5
	SL	17
Phonological output (N = 4)	TA	0
	SB	0
	AK	0
	SBS	2
Control group 7th grade: Average (SD) N		0.5 (0.79)
		18

Shaded cells denote a migration rate that is significantly higher than the control group using Crawford and Howell’s [66] *t*-test.

## Data Availability

The data presented in this study are available upon request from the corresponding author. The data are not publicly available due to ethical restrictions.

## Footnotes

1 Notice that the term “attentional dyslexia” is somewhat confusing. When Shallice and Warrington discovered this type of dyslexia, they termed it “attentional dyslexia” because they believed it results from a selective attention deficit [12]. However, today we know that this is an orthographic-specific deficit (possibly an attentional orthographic-specific deficit), rather than a general visuo-spatial attention disorder. Dissociations were reported between selective attention deficits and attentional dyslexia [15,16,17]. Possibly, a more accurate name for this dyslexia would be “between-word migration dyslexia” or “letter-to-word binding dyslexia”. Seeing how cumbersome these more accurate names are, the short and accepted name, though confusing, remains. Also notice that when we say “attentional dyslexia” here, we refer to a very specific type of dyslexia in the orthographic visual analyzer that causes between-word migrations. We do not refer to general claims that dyslexia (of all kinds) results from some sort of an attentional deficit [18,19,20,21]. Due to these considerations, in the following discussion we therefore mainly use the term “between-word migrations resulting from an orthographic input deficit”.

2 A deficit in word and nonword repetition could also arise from a deficit in the phonological input buffer, but a phonological input buffer deficit should not affect reading aloud, so it cannot explain our participants’ migrations in reading aloud. Therefore, we assume that impaired nonword and word repetition in our participants indicates that they have a phonological output buffer deficit.

3 Should a deficit in the phonological output buffer be called “dyslexia”, e.g., “phonological output buffer dyslexia”? If one defines dyslexia as a deficit in reading aloud, it is a kind of dyslexia. However, it is a deficit in the component that is not specific to reading, and as such may be thought of as a language deficit, which also affects reading aloud.
